# Perceived outcomes of music therapy with Body Tambura in end of life care – a qualitative pilot study

**DOI:** 10.1186/1472-684X-13-18

**Published:** 2014-04-07

**Authors:** Michael Teut, Cordula Dietrich, Bernhard Deutz, Nadine Mittring, Claudia M Witt

**Affiliations:** 1 Institute for Social Medicine, Epidemiology and Health Economics, Charité Universitätsmedizin Berlin, Luisenstr. 57, Berlin 10117, Germany; 2 Institute for Complementary and Integrative Medicine, University Hospital Zurich, Zurich, Switzerland; 3 Medical practice for psychotherapy, musictherapy and relaxationtherapy, Käthe-Niederkirchner-Straße 5, Berlin 10407, Germany; 4 Klangwerkstatt, Christburger Straße 31, Berlin 10405, Germany

## Abstract

**Background:**

In recent years, music therapy is increasingly used in palliative care. The aim of this pilot study was to record and describe the subjective experiences of patients and their relatives undergoing music therapy with a Body Tambura in a German hospice and to develop hypotheses for future studies.

**Methods:**

In a qualitative interview pilot study, data collection and analyses were performed according to the methodological framework of grounded theory. We included German-speaking patients, or relatives of patients, receiving end of life care in an inpatient hospice setting.

**Results:**

11 persons consisting of 8 patients (age range 51–82 years, 4 male and 4 female) and 3 relatives were treated and interviewed. All patients suffered from cancer in an advanced stage. The most often described subjective experiences were a relaxing and calming effect, sensations that the body feels lighter, and the generation of relaxing images and visualizations. Family members enjoyed listening to the music and felt more connected with the sick family member.

**Conclusion:**

Patient reported beneficial aspects. The small sample size could be seen as a limitation. Assessment instruments measuring relaxation, stress, quality of life and should be included in future quantitative studies.

## Background

In recent years music therapy has been used increasingly in palliative care, especially in treatment of cancer patients [1]. Throughout history and up to the present, music and medicine have been closely interrelated [2]. Music therapy has been defined as „the use of sounds and music within an evolving relationship between client/patient and therapist to support and develop physical, mental and social spiritual well-being” [3].

Research on the usage of music therapy in palliative care has seen a continuous growth within the last decade [2]. A recent Cochrane review on music therapy in cancer patients [4] aimed to compare the effects of music therapy or music medicine interventions with both standard care alone and standard care paired with other interventions. The review included all randomized controlled trials (RCTs) and quasi-randomized trials of music interventions aimed at improving psychological and physical outcomes in patients with cancer. A total of 30 trials with 1891 patients were included in the analysis. Seventeen trials used listening to pre-recorded music and 13 trials used music therapy interventions that actively engaged the patients. The results indicated that music interventions may have beneficial effects on anxiety, pain, mood, and quality of life in cancer patients. Music therapy also may have a small effect on heart rate, respiratory rate, and blood pressure. The authors could not draw any conclusions about the effect of music interventions on distress, body image, oxygen saturation level, immunologic functioning, spirituality, and communication outcomes because there were not enough trials focused on these aspects. However, most trials were at high risk of bias: Only 19 out of 30 trials used appropriate methods of randomization, only 16 trials reported allocation concealment, only four trials reported blinding of outcomes and in eight trials participant withdrawals were not reported. Altogether only one trial was considered to be of low risk of bias. Thus it was concluded that the results need to be interpreted with caution.

Music therapy has been shown to be well suited and applicable to, hospice settings [2,5,6]. One advantage of music therapy is that it is a non-pharmacological intervention. Compared to many pharmacological interventions it is safe and has minimal side effects. In addition, it can be used to improve the communication between patient, family and medical team; it can help the patient to cope with all aspects of the disease, improves physical, emotional, social and spiritual well-being and may help to control and reduce pain [1,2,6-10].

In recent years the Body Tambura, a new instrument in the field of music therapy inspired by the classical Indian Tanpura, has received increasing attention by German music therapists working in palliative care or with coma patients [11]. The original Indian Tanpura is a long-necked plucked lute with four to six wire strings: Plucking the strings in a regular pattern creates a base tone harmonic resonance, which is called *bordun* or drone function. The notes of Tanpura are not part of the melody itself, but support and sustain the melody by providing a colorful and dynamic harmonic resonance field of basic tones.

The aim of this qualitative pilot study was to record and describe the subjective therapeutic experiences of advanced stage cancer patients and their relatives undergoing music therapy with the Body Tambura in a German hospice setting and to develop hypotheses for future studies.

## Methods

### Design

A qualitative study was performed using open interview techniques. An open interview technique is considered an appropriate and valuable research methodology in palliative care settings, as it allows for the inclusion of patients who would otherwise be unable to participate in other study designs, e.g. filling out questionnaires in quantitative studies [12,13].

Data collection and analyses were based on the methodological concept of grounded theory [14,15]. The study was approved by the ethics committee of the Charité - Universitätsmedizin Berlin (EA 1/191/10, 01.09.2010).

### Patients

The pre-screening of patients for the study was carried out by the caregiving nurses in a stationary hospice in Berlin with 16 beds.

Patients and their relatives were invited to participate in an interview study on music therapy with the Body Tambura. If they were interested, the music therapists would inform them about the intervention and the procedures of the study.

Inclusion criteria were: receiving palliative care in the hospice, age ≥ 18 years and ability to speak fluent German. Dying patients were excluded as were patients unable to participate due to severe clinical symptoms, unable to talk, or those under legal guardianship.

After providing written informed consent, patients were enrolled in the study and the music therapy started immediately.

If family members or caregivers participated in the music therapy session they were interviewed as well.

### Intervention

**Figure 1:**
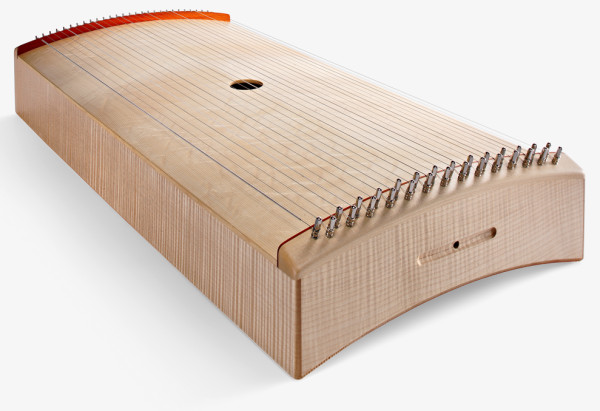
The Body Tambura.

The Body Tambura was designed as a therapeutical instrument to be placed on the human body (Figure 1). The initial motivation for this new development sprang from a music therapist’s request for an easy-to-handle body instrument for receptive music therapy work with bedridden patients (coma patients). The instrument consists of a very lightweight rectangular corpus (measuring 70 × 33 × 8 cm; L × W × H) equipped with an ergonomically contoured base and a sounding board fitted with 28 (i.e. 7 × 4) strings tuned in the same note pattern as the Indian Tanpura (A – d – d – D). The sound of the Body Tambura is characterized by playing the 28 strings of the instrument evenly to produce fine vibrations and create a softly enveloping monochromatic acoustic space for the listener, which is supposed to induce a state of trance and relaxation [7].

The recommended playing technique is a very even, confluent touching of the strings with the fingertips of both hands alternating. While playing, percussive sounds, background noise (fingernails) and mechanical vibrations/shocks caused by playing too hard should be avoided. In cross section the radius of its curvature is chosen such that it fits the contours of the human body without being constrictive. The contact surface is thus enlarged and can be placed over the patient like a cover. If the instrument is placed at the centre of an adult’s body, the length of the corpus creates contact points between the shoulders and the pelvic region. The corpus is made from fine-pored tonewood which guarantees good stability and optimal vibrational properties, both as a “listening” and as a “feeling” instrument. The instrument weighs roughly 2,200 g. Its construction – in terms of material thickness and quality - is such that the resonating chamber is fabricated to be as light as possible. However, a certain weight is required for good vibration transference and distribution over the entire upper body, which can provoke a pleasant experience.

The intervention was facilitated by two experienced music therapists, both having more than 10 years of professional experience. The Body Tambura was either placed directly onto the body of the patient while he or she was lying down or was played a short distance away from the patient. The duration of treatment was determined by the requirements of the patients. The intervention could be repeated in weekly sessions with up to five sessions for each patient. Each session began with a greeting followed by a short introduction and a request for feedback on the previous session. At the end of each session, the patients had the opportunity to give feedback and share their experiences.

### Interview guide and data collection

A semi-structured interview guide was developed by the researchers and music therapists based on former practical experiences and the study aims. Patients were asked:

● How did you experience the music therapy with the Body Tambura?

● What exactly has changed?

● What did you find enjoyable?

● Have you experienced any unpleasant feelings or emotions?

● Have your complaints changed? If so, can you specify which complaints and how they have changed?

● Would you recommend treating other people in your situation with this music therapy?

The main diagnoses and actual complaints of each patient were documented, in addition to basic sociodemographic information such as age, gender and educational background. The interviews were conducted in person in the hospice at the patients bedside by an independent researcher (medical doctor) a few hours after the sessions or on the following day. The aim was to conduct at least one interview with each patient and, should their health allow it, to conduct further interviews to explore their longitudinal experiences,. All participants gave their informed consent. Interviews were digitally recorded and the interviewer wrote up a short interview memo after each interview.

### Data analyses

All interviews were transcribed verbatim. Analyses followed a grounded theory approach assisted by the software MAXQDA® [16]. After the first four interviews were transcribed and analyzed, the next four interviews were conducted with taking into account new findings and questions developed from the first round of results. Data collection, coding and theory generation alternated, the analysis process occurred in a triadic and circular constant comparative manner [14,15]. Written memos during the coding and analysis process supported the analyses and results. The initial data analysis was performed by an experienced qualitative researcher and was critically reviewed by a peer researcher. Generated theories included in the results required the approval of both researchers.

## Results

We included 8 patients (4 male, 4 female) and two 3 relatives. Table 1 describes the patients’ age, gender, disease history and educational background. All patients suffered from cancer as the primary diagnosis, were terminally ill and were cared for in the participating hospice. All patients were at least able to participate in an interview at the time of the first intervention. In two cases, the possibility to communicate was restricted due to the disease progression, namely metastases in the brain, which resulted in disorientation.

**Table 1:** Patients’ age, gender, disease history and educational background

**Patient code**	**Age (years)**	**Gender**	**Disease history**	**Background**
*T1*	81	M	Prostate cancer, bone metastases	Retired
*T2 and husband*	51	F	Lung cancer, brain metastases	Housewife
*T3*	63	F	Brain tumor	Retired
*T4*	58	F	Hepatocellular carcinoma	Employee
*T5*	74	F	Lung cancer, metastases	Retired
*T6*	68	M	Colon cancer, metastases	Musician
*T7 and brother*	68	M	Bladder cancer, metastases	Nurse
*T8 and wife*	77	M	Lung cancer, metastases	Mason

The duration of the intervention was between 5 minutes up to half an hour.

The duration of the interviews was between 3 and 13 minutes (mean 7 ± SD 3 minutes), depending on the patients’ ability to communicate.

In most treatments, the Body Tambura was first played from a distance and then put on the body of the patient during the treatment. Most of the patients reported the close contact of the vibrating instrument as a pleasant experience. One patient compared the feeling of the instrument to a big pillow; others found the vibration of the instrument nice and slight, but the vibration was not always felt on the body. One interview participant reported that he observed an agitated reaction of his wife when the instrument was put on her body during the treatment. When asked about their subjective experiences all patients described the treatment in a positive way. Most patients would also recommend the therapy to other patients in a palliative care setting.

The most important described subjective experiences were feelings of relaxation, perceptions of changed body sensations and the provocation of pleasurable images or visualizations.

### Feelings of relaxation

Most patients described a relaxing and calming experience with the Body Tambura:

It [the treatment] was very nice, very relaxed. (…) I found it very comfortable and gorgeous. (T4)

One patient described, that the music therapy enabled him to relax after experiencing a very stressful time in various hospitals after undergoing several cancer therapies:

Yes, somehow I have become calmer. I’ve got very, … yes very exciting thing behind me and, uh - … - that’s why I say it has a calming effect on me …(T1)

### Changes in perceived body sensations

When asked about what exactly changed while experiencing the music therapy many patients explained, that they experienced a change in their body sensations. Most often this experience was described as a feeling of “lightness”, or “as if floating in the air”. One patient compared the treatment session to the relaxing effects of autogenic training he had experienced in the past.

As if you take off a little bit. As if you are floating a little bit, that is as if you do hear a good song and you close your eyes … as if you take off a little bit. (T4)

Well it has definitely loosened up … the body … one feels somehow a bit lighter. (T8)

### Images or visualizations

While experiencing the intervention patients described experiencing pleasant images and visualizations like “being in other spheres”, “swimming on waves” or hearing nice voices. Most often these images were connected to former experiences of relaxation that patients had encountered in the past or to symbolic situations associated with feelings of calmness and inner peace.

Well, it (…) feels somehow like swimming on waves, where you feel good (…). (T1)

Yes, that is what I meant: angel harps. Angels. (T4)

I have imagined myself lying on the beach: Sun, … and … yes hear the sound of the sea and - … - see dolphins jumping around somehow (laughs easily). (Family member of Patient T 7)

### Connecting to family

For some of the patients it was also very important to tell the interviewer how their family members felt during the session and that they also liked the treatment very much. A shared positive experience of the music therapy seemed to facilitate a connection between the patients and the family members. During some interviews, family members of patients were present and thus asked to share their experiences of the intervention. The family members described their own experiences of the music. They were very enthusiastic about the therapy and found it comforting. Furthermore, it had calming and relaxing effect on them and it was associated with other types of experiences such as autogenic training or being at the beach.

One family member even reported that he felt a relief of muscle tension and pain due to the treatment. The family members also observed a calming and relaxing effect on their relatives.

One relative mentioned that the experience of the treatment session could help her to turn her sorrow about the disease into gratitude for being able to spend time with her family member.

And somehow (…) my sorrow, that I don’t want to rationalize away, has a bit turned into (…) gratitude for being able to be here. (…) This felt very, very good. (Family member of Patient T 7)

## Discussion

The most often described subjective experiences of patients were relaxing and calming effects, sensations that the body feels lighter, and the provocation of peaceful images or visualizations. Family members enjoyed listening to the music and in doing so felt more connected with their sick family members. Seen in the context of the growing body of studies on music therapy in palliative and cancer care this study is the first to explore therapeutic experiences with the Body Tambura in a stationary hospice setting.

Patients included in this study suffered from cancer and were in a progressed stage of end of life care. They were not able to participate in the interviews for more than 5 to 15 minutes. Furthermore, the ability to explain experiences was limited, especially in those patients with brain metastasis. Although the verbal communication was restricted to a few minutes, the data was homogeneous enough to see a consistent picture that highlights that patients and their relatives felt more relaxed, experienced pleasant images and experienced a change of body sensations.

A clear limitation is the fact that the sample is relatively small and we did not use a control group for comparison. Therefore, our results must be interpreted with caution and should be used to develop hypotheses for a larger trial combining qualitative and quantitative research. The four themes “feeling of relaxation”, changes in body sensation”, “images and visualizations” and “connecting to family” could clearly be coded and summarized from the patients’ narrations. However, the data did not allow us to generate further hypotheses regarding other treatment experiences. We believe that our data covers the most important experiences, but of course, the inclusion of more patients in future trials might reveal additional relevant experiences.

It is important to understand, that the applied music therapy was fully passive and receptive. Active participation of the patient (e.g. singing, playing an instrument) was not necessary. Given the fact that the Body Tambura is easily applied, the therapy itself is very well suited for the needs of patients in hospice settings. The nursing staff of the Berlin hospice asked to be trained in playing the Body Tambura after completing the study and were taught how to apply the instrument in a weekend workshop. Treatments with the Body Tambura are now regularly offered in the hospice by nurses and are very popular.

From this qualitative data we were able to generate the following hypothesis: Music therapy with the Body Tambura might help end of life patients to relax, to feel positive body sensations, to have positive images or visualizations, and to connect with their families and friends while sharing the experience of the instruments’ sound. In future quantitative confirmatory studies, simple measurements assessing relaxation, stress, quality of life and well-being could be used and a routine therapy group or another active treatment group such as therapeutic touch or empathic listening could be used as a comparison.

It is worthy of note that the reported positive effects of the Body Tambura could have been a result of the music itself, the fact that someone was attending the patient, the therapeutic relationship or the expectations generated by the therapist’ explanations of the therapy [9], or most likely a combination of such aspects. However, what remains paramount and unique to the Body Tambura is the harmonic acoustic space created by the instrument and the vibrations felt on the patients’ body. Both, acoustic and sensory stimulation in combination with the suggestions of the music therapists might be able to induce a trance like state of relaxation comparable to hypnotic relaxation [17]. This hypothesis is supported by the research of Zeigert [18] who reviewed the existing therapeutic evidence and experience with the Body Tambura. According to his research, the intervention could be understood as a form of vibroacoustic therapy that combines hearing (auditory perception) with the perception of vibrations (somatosensoric perception of pressure, touch and vibration) that may induce a state of kinesthetic trance and relaxation.

Although this was reported in other studies we were not able to determine if the Body Tambura had a positive influence on pain, as our patients did not suffer from pain at the time the intervention was delivered. One explanation for this phenomenon might be that the hospice staff selected only patients to participate who were not suffering from pain at the time of the study.

## Conclusion

Palliative care patients in a hospice setting treated with the Body tambura reported relaxing and calming effects, sensations that the body feels lighter, and the provocation of pleasant images or visualizations. Family members enjoyed listening to the music and felt more connected with the sick family member after the music therapy.

Quantitative studies to evaluate the effectiveness would be a good next step. Measures for relaxation, stress, quality of life and wellbeing should be included in future investigations.

## Competing interests

Bernhard Deutz is the developer and manufacturer of Body Tamburas, Cordula Dietrich runs courses on music therapy and teaches the therapeutic use of the Body Tambura in palliative care settings. All other authors declare that they have no competing interests.

## Authors’ contributions

MT, CD, BD and CW designed the study. CD and BD treated the patients. MT collected the data. NM and MT and analyzed the data, MT, NM and CW prepared the manuscript. CW and MT had the overall responsibility. All authors were involved in interpreting the results of the analyses and critically reviewed the manuscript. The final version was approved by all authors.

## Pre-publication history

The pre-publication history for this paper can be accessed here:

http://www.biomedcentral.com/1472-684X/13/18/prepub
